# Supplementary material for: Influence of Surface Corona Discharge Process on Functional and Antioxidant Properties of Bio-Active Coating Applied onto PLA Films
Source: Antioxidants (Basel). 2023 Apr 1;12(4):859. doi: 10.3390/antiox12040859 (PMC10135253; doi:10.3390/antiox12040859)
Supplement: Supplementary file 1 [file antioxidants-12-00859-s001.zip › Supplementary data - Nativia NTSS - EU.pdf]

NATIVIA® NTSS

BoPLA transparent film, both sides heat sealable, biodegradable

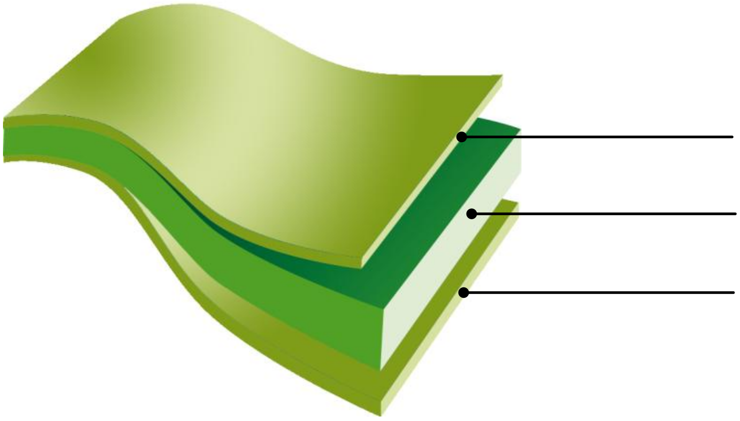

Heat sealable PLA-layer (MST= 85°C)

PLA core

Heat sealable PLA-layer (MST= 85°C)

ENVIRONMENTAL

- Made from annually renewable source
- Biodegradable into carbon dioxide, water and biomass by microbial digestion
- Certified DIN EN 13432 (7H0052) for Compostable intermediates.

This logo can be used for final products which are made of this intermediate and certified with a 7P-number as final products at DIN CERTCO.

compostable

- Certified TÜV AUSTRIA (four-star certification) OK biobased (S206)

OK biobased TÜV AUSTRIA S0206

PROPERTIES

- Heat sealable on both sides
- Good mechanical properties
- High stiffness
- Good oxygen barrier
- Excellent moisture transmission
- Good slip properties
- Resistant to oil, fat and alcohol
- Excellent twist retention

TYPICAL APPLICATIONS

- Rotogravure and flexographic printing
- Lamination
- Single web structure
- HFFS and VFFS packaging
- Twist and Dead Fold
- General wrapping

ROLL SIZE AVAILABILITY*

Film	Standard Length (m)	4x ** (m)	7x (m)
NATIVIA® NTSS 17	3,750	15,000	26,250
NATIVIA® NTSS 20	3,200	12,800	22,400
NATIVIA® NTSS 25	2,550	10,200	17,850
NATIVIA® NTSS 30	2,100	8,400	14,700
NATIVIA® NTSS 35	1,800	7,200	12,600
NATIVIA® NTSS 40	1,600	6,400	11,200
NATIVIA® NTSS 50	1,300	5,200	9,100
Outside diameter – core 76 mm	305 mm	588 mm	
Outside diameter - core 152 mm	337 mm	605 mm	783 mm

*Regional availability of roll sizes (multiples of standard length)-please refer to the corresponding Sales Representative
**Length rolls on 152 mm cores could be produced with reduced length in Europe to maintain roll diameters below 600 mm

NATIVIA® NTSS

Properties	Method	Unit	Ref.	Typical values						
Nominal thickness		µm		17	20	25	30	35	40	50
Unit weight	Internal method	g/m²		21.1	24.8	31.0	37.2	43.4	49.6	62.0
Yield		m²/kg		47.4	40.3	32.3	26.9	23.0	20.2	16.1
Tensile strength	ASTM D882	N/mm²	MD<br>TD	105<br>205						
Elongation at break		%	MD<br>TD	185<br>85						
Dynamic COF	ASTM D1894		INT/INT	0.35						
Haze	ASTM D1003	%		1.0	1.5			1.8		
Gloss (45°)	ASTM D2457	Gloss Unit		80						
Heat seal range	Internal method	°C		85 - 140						
Seal strength	Internal method<br>85°C ;0.5 s	g/cm		230		290	350			
Treatment Level	ASTM D2578	mN/m		37						
Water vapour permeability	ASTM F1249<br>(38°C - 90% RH)	g/m²/d		550	440	330	270	230	200	170
Oxygen permeability	ASTMD3985<br>(23°C - 0% RH)	cm³/m²/d		1300	1100	900	730	630	540	430

Order volume tolerance		
	≤ 1.000 kg	± 20%
Weight	1.001-10.000 kg	± 10%
	> 10.000 kg	± 5%

PRINTABILITY/LAMINATION

NATIVIA® can be converted on flexo- and rotogravure print presses. In comparison to other polyolefin substrates, it needs rather low drying temperatures and a high airflow for best print results. Any solvent, except Ethyl Acetate, can be used. Ethyl Acetate will cause swelling effects up to total disintegration of the PLA. It is important to contact your ink and/or adhesive supplier for best choice of products. Appropriated tests should be carried out before converting.

STORAGE, HANDLING AND APPLICATION

NATIVIA® NTSS does not require special storage conditions. A storage temperature below 30°C is needed in order to minimise the deterioration of the film properties in general. It is advisable to turn over the inventory according to the delivery date (first in - first out). The film should be conditioned in the operating environment at least for 24 hours before processing. NATIVIA® NTSS is suitable for use up to 6 months from the date of production, if properly stored.

INDICATION OF SURFACE TREATMENT

NATIVIA® NTSS is presenting a natural surface tension of 37 mN/m on both sides without decay over time.
NATIVIA® NTSS can be supplied either with additional corona treatment on the outside surface (TO) or on the inside surface (TI). This must be agreed with our sales representative before processing the order.

FOOD CONTACT

NATIVIA® NTSS complies with EU and FDA regulations. Specific documents and MSDS are available on request.

The property values represented in the table do not constitute product specifications, but represent the average or typical values. Use of this information is limited to the specific recipient. While the information is accurate to the best of our knowledge as of the date compiled, it is limited to the information as specified. No representation or warranty, expressed or implied, is made regarding the information, or its completeness or fitness to a particular use. The user is solely responsible for all determinations regarding use and we disclaim liability for any loss or damage that may occur from the use of this information. *Ti* does not guarantee the typical (or other) values.
